# Delayed spinal arachnoiditis with syringomyelia following aneurysmal subarachnoid haemorrhage: a case report with patient experience

**DOI:** 10.1038/s41394-024-00654-1

**Published:** 2024-06-10

**Authors:** Nityanand Jain, Liga Jaunozolina, Inga Putraima, Kaspars Auslands, Andrejs Millers

**Affiliations:** 1 Faculty of Medicine, Riga Stradinš University, 16 Dzirciema Street, Riga, LV-1007 Latvia; 2 Children Clinical University Hospital, Vienības Gatve 45, Riga, LV-1064 Latvia; 3 Department of Neurosurgery, Riga East Clinical University Hospital, 2 Hipokrata Street, Riga, LV-1038 Latvia; 4 Department of Neurology and Neurosurgery, Riga Stradinš University, 16 Dzirciema Street, Riga, LV-1007 Latvia

**Keywords:** Spinal cord diseases, Headache

## Abstract

**Background and importance:**

Syringomyelia, or the formation of fluid-filled cysts within the spinal cord, associated with delayed spinal arachnoiditis is an uncommon complication of aneurysmal subarachnoid haemorrhage. To date, about 18 cases have been reported in medical literature, with just two reported in patients under the age of 35 years.

**Clinical presentation:**

A 27-year-old female patient complained of sudden, severe headaches in the occipital region, nuchal rigidity, and drowsiness when she presented at our institution. A head computed tomography scan revealed intraventricular bleeding in the lateral and fourth ventricles with more extensive haemorrhaging in the frontal horns. A left posterior inferior cerebellar artery (PICA) aneurysm was confirmed via digital subtraction angiogram, and endovascular embolization was done. Two years later, the patient reported intense pain in the lower back along with symptoms suggestive of spinal cord compression. Spinal magnetic resonance imaging (MRI) showed spinal adhesions from C1 to L4, syringomyelia with some vasogenic oedema extending from T3 to T9 level, and a cyst in the lumbar region. Consequently, a right hemilaminectomy was performed along with microsurgical release of arachnoid adhesions and placement of a subdural drain. Radiological and symptomatic improvements were observed. Since then, the patient’s clinical condition has remained stable during the past three years of follow-up visits.

**Conclusions:**

Literature on optimal treatment modalities and patient prognosis is scarce and debated. The time for symptom improvement depends on the level and extent of spinal cord involvement. Rehabilitation may be required for most patients, as complete symptomatic recovery may not be attainable.

## Background and importance

Aneurysmal subarachnoid haemorrhage (aSAH) accounts for ~85% of all subarachnoid haemorrhages and is linked to a high early mortality rate [1, 2]. It has a global incidence of 7.9 per 100,000 person-years, with females at a higher relative risk [3]. Other significant risk factors include age (>60 years), high blood pressure, smoking, alcohol use, oestrogen deficient status, and family history [2, 4]. A rare complication of aSAH is the development of delayed spinal arachnoiditis, which is characterized as persistent inflammation in the arachnoid mater that leads to scarring and nerve root tethering [5]. Spinal arachnoiditis appears to have a post-infectious pathoetiology, particularly in patients with compromised immune systems [6]. In isolated cases, spinal arachnoiditis has been reported to be associated with syringomyelia, the formation of fluid-filled cysts called syrinxes within the spinal cord.

Because of the rarity of the condition, knowledge of its clinical presentation, pathogenesis, management, and long-term outcomes is limited. In reviewing the literature, we were able to identify 18 cases with syringomyelia post-aSAH that were reported between 1940 and 2023 (Table 1) [7–21]. All patients were above 45 years old, with two exceptions aged 22 and 35 years [7, 20]. Among the reported cases, seven patients were male and the most frequent cause of SAH was an aneurysm of the posterior inferior cerebellar artery (PICA). The latency period between surgical management of SAH and diagnosis of syringomyelia ranged from two weeks to 11 years. Patient follow-up period ranged from four months to six years. Herein, we present an illustrative case report of a young adult patient without known risk factors for aSAH who experienced delayed spinal arachnoiditis in the cervical region concomitant with syringomyelia in the upper thoracic spine. Additionally, we present the patient’s long-term follow-up, including her perspective on quality-of-life issues.

**Table 1:** Overview of the case reports reported in the literature with syringomyelia post-aneurysmal subarachnoid haemorrhage.

Study	Country	Age (y)	Sex	Aneurysm location	Symptoms	Latency	Syrinx (cyst) Location	Treatment of Syrinx	Follow-up time	Reference
Lorenzana-Honrado et al., 1996	Spain	22	F	Left PICA	Progressive sciatic pain, urinary urgency	Sometime after 2 weeks	T6 to L4	External cyst drainage with shunting	1.5 years	[7]
Taguchi et al., 1996	Japan	59	M	Left VA	Gait imbalance, loss of balance, bilateral foot numbness	3 months	T5 to T8	Laminectomy with cyst puncture	1 year	[8]
Tumialan et al., 2005	USA	53	F	Left PICA	Gait disturbances decreased left LE proprioception	1 year	C7 to T3; multiple loculated arachnoid cysts	Laminectomy	7 months	[9]
Marshman et al., 2007	UK	57	F	Left PICA	Gait disturbances, hyperreflexia	2 years	T1 to T10; multiloculated cystic lesion	Laminoplasty	1 year	[10]
Eneling et al., 2011	Sweden	57	F	ACA	Gait ataxia, sensory disturbances, bladder and bowel issues	18 months	T4 to T8; post-laminectomy extension to L1 within 8 months	Laminectomy with shunting	Not reported	[11]
	Sweden	65	F	Left PICA	Tetraparesis, bladder and bowel issues	8 years	Cervical to midthoracic region; Extension to conus within two years without surgery	Laminectomy with shunting	2 years	
Abel et al., 2012	USA	66	M	Right VA	Neck pain	11 years	C1 to mid-thorax	Laminectomy with adhesion microlysis and shunting	2 years	[12]
	USA	48	F	Right SCA	LE weakness	8 years	C4 to conus	Laminectomy with adhesion microlysis and shunting	2 years	
Abhinav et al., 2012	UK	58	F	Left PICA	Gait disturbances, LE weakness, and paraesthesia	9 months	Lower cervical and thoracic region; recurrence in the same region after 1 year	Shunting with drainage of cyst; on recurrence hemilaminectomy, septostomy and shunting	6 years	[13]
Ishizaka et al., 2012	Japan	63	F	BA	Gait disturbances, LE numbness, urinary incontinence	3 years	T2 to T11; multiple cystic lesions on the ventral side in thoracic region	Laminectomy with adhesion microlysis without shunting; cyst puncture	3 years	[14]
Nakanishi et al., 2012	Japan	54	F	Left MCA	Back pain and numbness, gait disturbances	20 months	C7 to T10 level	Laminectomy and durotomy with shunting	5 years	[15]
	Japan	49	M	Left VA	Headache, diplopia, hoarseness, ataxia, dysphagia	5 months	Medulla oblongata to C6 level; Arachnoid cyst—cisterna magna	Foramen magnum decompression and laminectomy with shunting	2 years	
Rahmathulla and Kamian, 2014	USA	54	F	Right PICA	Hypaesthesia around lips and LE spasticity	1 year	C1 to C2; C5 to C6; T4 to T5; multiple cysts	Laminectomy with adhesion microlysis and dissection	18 months	[16]
McAlpine and Adamides, 2016	Australia	70	F	ACA	No complains	13 days	C3 to T2	Observation (patient improved and resumed day-to-day activities)	17 months	[17]
Davidoff et al., 2017	Australia	66	M	PICA	LE sensory issues and weakness, diplopia, sleep apnoea	32 months	C1 to T1	Posterior fossa decompression, division of adhesions, pericranial graft duraplasty, and shunting	27 months	[18]
Machida et al., 2018	Japan	49	M	Right VA and PICA	Tetraplegia	13 days	C1 to T4	External ventricular drainage and removal of lumbar drain	2 years	[19]
Huang et al., 2020	China	35	M	Right VA	Left sided weakness and shoulder pain	6 months	C1 to C7	Rehabilitation programme	4 months	[20]
Nagashima et al., 2022	Japan	66	M	VA	Gait disturbances and urinary issues	11 years	T5 to T8	Laminectomy with duraplasty	Not reported	[21]
Jain et al., 2024	Latvia	27	F	Left PICA	Severe lower back pain, LE weakness, and loss of deep sensations	2 years	Starting from T3-T4 and extending till T9	Right hemilaminectomy with adhesion microlysis and shunting	4 years	Present case

Studies were identified using a search based on Google Scholar and PubMed followed by forward and backward citation search. Search terms—(“Spinal Syringomyelia” OR “syrinx” OR “spinal arachnoid cyst”) and (“subarachnoid haemorrhage” OR “subarachnoid haemorrhage”) AND (“case report” OR “case” OR “patient”). Search was conducted on 25th November 2023.

*ACA* anterior communicating artery, *BA* basilar artery, *LE* lower extremity, *MCA* middle cerebral artery, *PICA* posterior inferior cerebellar artery, *SCA* superior cerebellar artery, *VA* vertebral artery.

## Clinical presentation

A 27-year-old female patient, with no prior history of serious illness, presented to the ambulance during the night with sudden-onset severe headaches in the occipital region while sleeping, nuchal rigidity when moving the head, drowsiness, and two episodes of vomiting. The patient, working as a nurse, reported no prior medication use, alcohol consumption, smoking habit, or use of narcotics. At the time of presentation to the ambulance, her vital signs were stable (BP—120/80 mmHg; pulse—90 beats per minute; respiratory rate—16 breaths per minute; and Glasgow Coma Scale GCS—15 points). The patient received an intravenous 2 ml bolus of 500 mg/ml Metamizole for pain management, intravenous 2 ml bolus of 10 mg/2 ml Metoclopramide as an antiemetic, and saline for fluid replenishment.

**Fig. 1:**
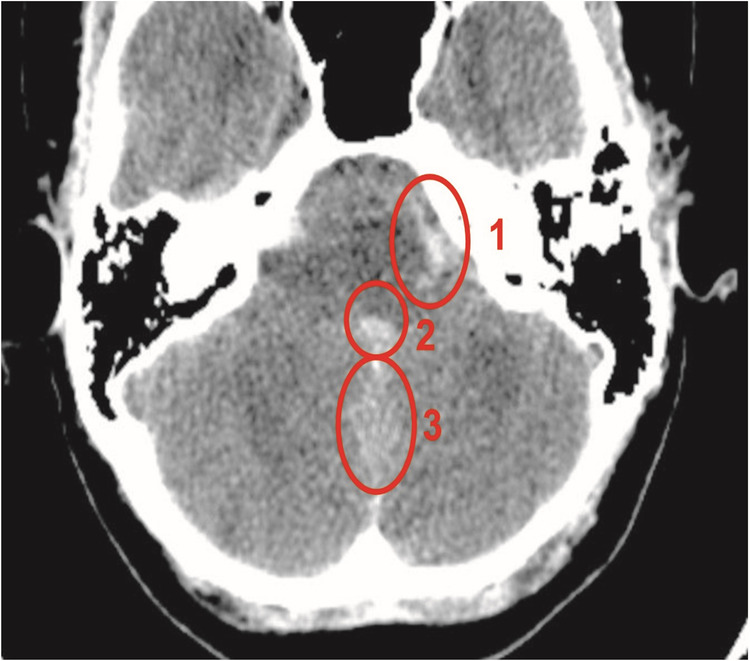
A non-contrast CT head without contrast demonstrating spontaneous subarachnoid haemorrhage (SAH) in the basal cisterns (circle 1), in the fourth ventricle (circle 2), and along the cerebellar hemispheres (circle 3) caused by ruptured left posterior inferior cerebellar artery (PICA) aneurysm.

**Fig. 2:**
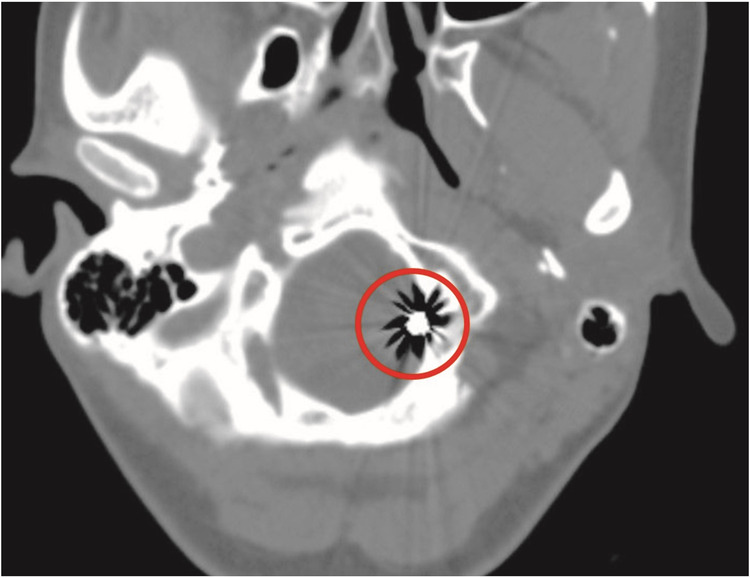
Postoperative non-contrast CT head demonstrating left posterior inferior cerebellar artery (PICA) aneurysm filled with coils (red circle).

A CT scan of the head without contrast was done and showed intraventricular bleeding in the lateral and fourth ventricles, with more extensive bleeding in the frontal horns. Additionally, bleeding was found around the foramen magnum that extended into the spinal canal (Fig. 1). The on-call radiologist recommended a magnetic resonance imaging (MRI) scan to eliminate differential diagnoses including malignant lesions and arteriovenous malformations. Clinically, the patient rapidly deteriorated and experienced a decline in consciousness within one hour, registering a GCS score of nine points. Consequently, the patient was transferred to the intensive care unit and underwent a ventriculostomy. The following morning, the patient was electively intubated, and a digital subtraction angiography (DSA) was performed, revealing an aneurysm of the left PICA in the P1-P2 segment (Fig. 2). The patient was prescribed Tab. Nimodipine 60 mg six times a day.

The left PICA aneurysm was successfully treated through endovascular embolization. An intraoperative CT scan was done to rule out any procedural complications. The CT revealed an enlarged ventricular system, diffuse subarachnoid haemorrhage (Fisher IV), and intraventricular haemorrhage. The patient was extubated during the day with complaints of headache. Three days later the patient complained of increasing intensity of headache, nuchal rigidity, and became bradycardic (pulse 56 bpm). Another CT head scan was performed, revealing infratentorial and supratentorial oedema, subarachnoid bleeding, and signs of haemorrhage in the cerebral aqueduct and fourth ventricle. Consequently, the drainage rate of cerebrospinal fluid (CSF) was increased to decrease the oedema. A check CT taken a week later showed residual blood in the ventricles and SAH spaces without ischaemic complications or hydrocephalus. Consequently, the ventriculostomy was evacuated after being in place for about ten days.

The patient was transferred to the semi-intensive neurosurgery ward with a GCS score of 15, no focal deficits, and mild nuchal rigidity. Three days later, the patient reported feeling drowsy, sleepy, and experiencing increased nuchal rigidity, resulting in a GCS score of fourteen points. No changes were seen in the CT head scan. A lumbar puncture was performed at the L3-L4 level, and the purulent CSF so obtained was found to be positive for *Klebsiella pneumoniae*. The bacteria were sensitive to meropenem. Based on the recommendation of the infectologist, intravenous meropenem 2 g was started thrice daily for six weeks due to suspected secondary meningitis and ventriculitis. The patient’s condition remained stable for the following week with signs of infection reduction as shown by repeated lumbar punctures.

A physiotherapist examined the patient and reported symmetrical muscle strength, with no signs of paresis or sensory deficits. The patient was fully independent and able to walk outside of the ward. However, the physiotherapist observed slight coordination deficits and emotional disturbances, along with right-sided paresis of the abducens nerve as confirmed by the ophthalmologist’s consultation. Two weeks later, the patient was discharged from the hospital with a referral to undergo DSA in three months and to enrol in a rehabilitation centre. At the time of discharge, the patient complained of mild pain in the lumbar region, though, we did not observe any paresis or meningeal signs. Four months later, the patient underwent the follow-up DSA, revealing full occlusion of the PICA aneurysm (Montreal A). No cognitive changes were observed (GCS 15 points), and no signs of abducens nerve palsy were noted. The patient was instructed to return for another DSA after one year. The subsequent follow-up DSA confirmed stable full occlusion of the aneurysm.

### Re-hospitalization due to delayed spinal arachnoiditis and syringomyelia

**Fig. 3:**
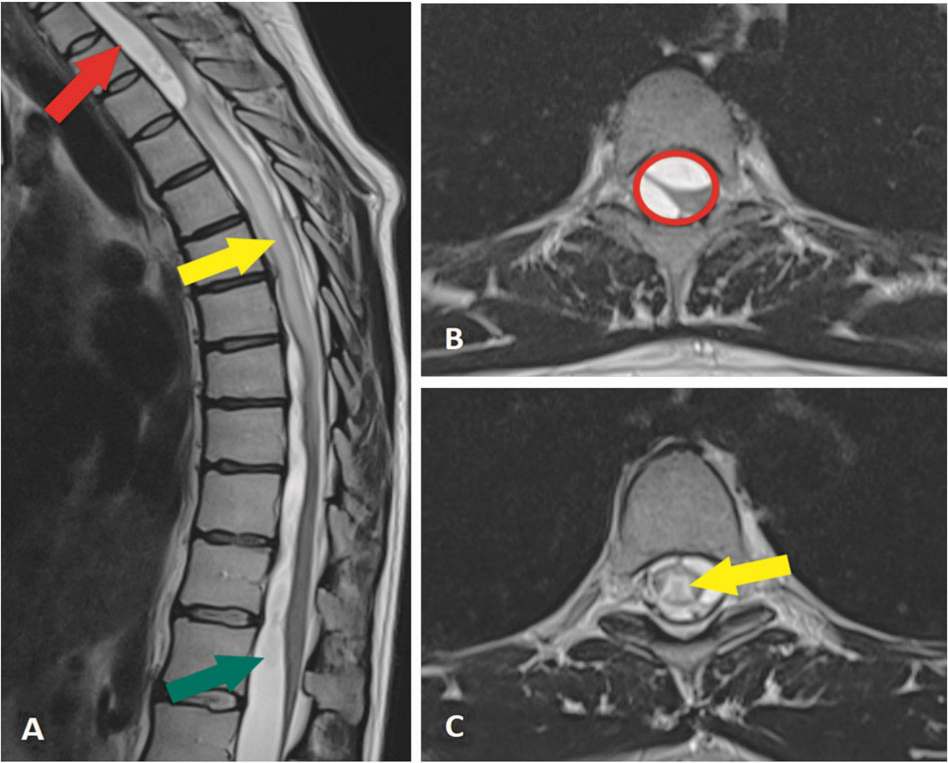
Preoperative T2 sequence MRI images in the (**A**) sagittal and (**B**) axial axis of thoracic spine showing dural adhesions (red circle) and septations, more pronounced at T2-T3 level, with arachnoid cysts (red and green arrows) above and below this level; (**C**) Spinal cord deformity and dislocation, with slightly widened central canal suggesting syringomyelia (yellow arrow) and some vasogenic oedema in spinal cord central parts.

However, several months later, the patient reported severe lower back pain and symptoms of spinal cord compression, including weakened muscle strength (particularly on the right side), slight clumsiness in the right leg for six months, and loss of deep sensations, including proprioception, in the legs. The patient also experienced difficulties in beginning and maintaining urinary stream and loss of temperature sensation in the lower body. The patient underwent an MRI scan that revealed a SAH bleed and bacterial meningitis complications (spinal adhesions) from C1-L4. Syringomyelia with some vasogenic oedema and spinal deformity were observed extending from T3 to T9 level with maximal deformity in the T3-T4 region. A cyst was also visualized in the lumbar region (Fig. 3). The Babinski sign was found to be positive.

**Fig. 4:**
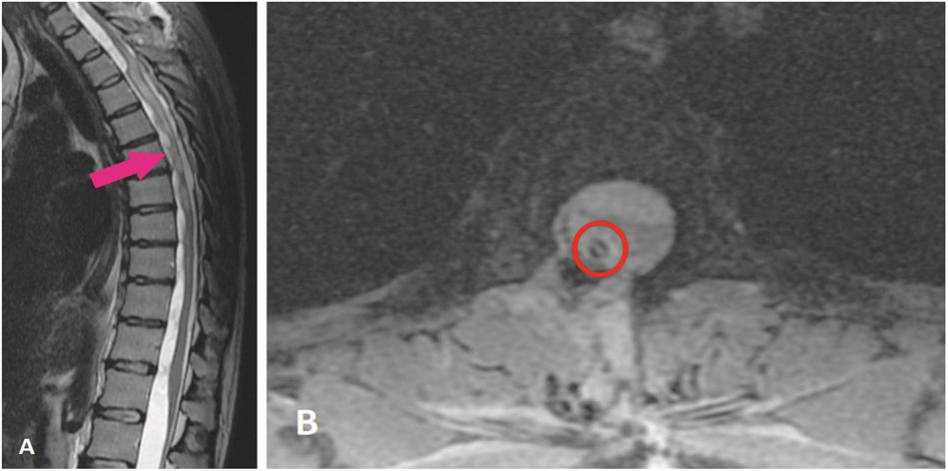
Postoperative T2 sequence MRI images in the (**A**) sagittal axis and (**B**) T2 merge axial images after right sided laminotomy, arachnoidolysis, and placement of a cysto-subdural shunt (red circle) at the T2-T3 level posterior to the spinal cord. Improvement of CSF circulation and marked reduction of spinal cord oedema can be visualized (pink arrow).

Accordingly, a right hemilaminectomy was performed at the T3-T4 level with microsurgical release of arachnoid adhesions. The patient requested to postpone the lumbar cyst drainage procedure. A subdural drain was then inserted to prevent adhesions from blocking CSF flow through the spinal canal. MRI showed improvement in CSF circulation and marked reduction of spinal cord oedema (Fig. 4). Post-operative consultation with the physiotherapist showed unstable gait and decreased muscle strength on the right side. Although voiding dysfunction persisted, the patient was able to walk unassisted. The patient was discharged from the hospital after nine days with mild neurological deficit and a referral to a rehabilitation centre. Three months later, a follow-up DSA was conducted, which showed no changes in the findings—full occlusion of the aneurysm (Montreal A). Since then, patient’s clinical and radiological status has remained unchanged for the past three years during follow-up visits.

### Patient perspective

About a year and a half after experiencing SAH with an aneurysm rupture and bacterial meningitis, I began feeling a sense of weightiness in my right leg. I became clumsier, my gait became unsteady, and I experienced a change in temperature sensation. It progressively became more challenging to engage in physical activities such as running and sports. I also observed issues with the pelvic organ functions, such as difficulty emptying my bladder and decreased sensation in the perineal area. I did various MRIs and other functional exams to establish the diagnosis. After consulting with a few neurologists, the final diagnosis of spinal cord compression was determined, accompanied by spinal cord oedema and spinal arachnoiditis with arachnoid cysts in the spinal canal.

Currently, after surgery and multiple physiotherapy and rehabilitation sessions, I feel an increase in the load tolerance and muscle strength in my right leg. However, significant balance disorders and muscle spasms persist. I continue to experience a loss of sensation, particularly in my foot, leading to frequent falls. Additionally, I feel uncomfortable when sitting for prolonged periods of time. Also, functional disorders of the pelvic organs persist - including neurogenic bladder, bowel dysfunction, and sexual dysfunction—which disrupt daily life and emotional well-being. I do engage in physical activity such as walking, running, and stretching exercises, as well as deep breathing. According to the Adhesive Arachnoiditis classification developed by the Tennant Foundation, I am categorized as Stage II (moderate) [22]. I maintain a balanced diet and supplement it with nutrients such as curcumin, vitamins C and B12, choline and inositol, and magnesium.

## Discussion

In our patient, it is plausible to suggest that both aSAH (non-infectious aetiology) and *Klebsiella pneumoniae* infection (infectious aetiology) may have contributed to the development of spinal arachnoiditis and syringomyelia. Although the pathophysiology underlying the development of spinal arachnoiditis following aSAH remains to be determined, it is hypothesized that the haemorrhagic inflammatory response to aneurysmal rupture plays a pivotal role. This process, initiated at the rupture site, continues through the fibroproliferative cascade, leading to the development of arachnoiditis at any point along the neural axis [15].

With regard to the infectious aetiology of post-meningitis spinal arachnoiditis and syringomyelia, several bacterial, fungal, and viral agents have been described in the literature. The most frequently reported pathogen appears to be *Mycobacterium tuberculosis* [5, 23–25], followed by isolated cases of pathogens such as *Listeria* and *Candida* [26, 27]. In the context of *Klebsiella* infections, while meningitis has been routinely described in the literature as a post-neurosurgical nosocomial infection, other central nervous system infections have been observed to be exceedingly rare. Thus far, we have been unable to find a documented case of *Klebsiella* meningitis progressing to spinal arachnoiditis and syringomyelia.

It is possible that spinal arachnoiditis may develop because of persistent aseptic inflammation in individuals who have previously developed meningitis [28]. The associated leptomeningeal scarring could trigger a delayed inflammatory fibroproliferative reaction resulting in fibrino-collagenous exudate that adheres the nerve roots to either themselves or the thecal sac (adhesive arachnoiditis) [28, 29]. These scarring changes can further induce thrombotic changes in the meningeal and spinal cord vessels, obstructing vascular flow to the spinal cord and resulting in focal ischaemia and necrosis [23]. Alternatively, the scar can block the communication between the subdural and subarachnoid spaces, thereby shortening the length of the subarachnoid space and impairing the CSF flow dynamics. This attenuates the ability of the spinal theca to absorb the subarachnoid CSF pressure waves that act on the spinal cord above the scar [30], driving the CSF into these spaces and eventually into the central canal, leading to syrinx formation [5, 31, 32].

In adhesive arachnoiditis, it has been postulated that the scarring also exerts an outward pressure on the spinal cord, causing enlargement of the central canal and creation of a negative suction pressure, which in turn leads to accumulation of fluid within the central canal [32]. The sustained drainage of the syrinx can result from several different sources, including the disrupted tissue planes (due to shearing stress from scarred meninges) and venous congestion of supplying vessels [33]. Obstruction and subsequent expansion of the perivascular Virchow-Robin spaces (due to compression of capillary circulation by dilated parenchyma) may result in the formation of small pools of extracellular fluid that drain into the syrinx [34, 35]. Additionally, unabsorbed subarachnoid pressure waves exert compressive force on the spinal cord, pushing more CSF into the syrinx [30]. Lastly, the compression of the capillaries under force reduces the absorptive surface area, limiting capillary capacity to remove CSF from the syrinx [36].

The primary treatment reported in literature for syringomyelia associated with arachnoiditis is the laminectomy procedure accompanied by adhesion microlysis and/or insertion of a shunt. It has been demonstrated that surgical removal of the obstruction has the potential to reverse the clinical symptoms and to provide long-term resolution [30]. However, the extent to which these symptoms are alleviated is directly proportional to the number of spinal segments affected, with involvement of four or more spinal segments necessitating shunting [30]. Shunting CSF to the subarachnoid, pleural, or peritoneal space enables unobstructed and continuous flow, eliminating the necessity for multiple cystic drainages while also producing sustained enhancements in neurological complications [37, 38]. Nonetheless, the efficacy of shunting has been a topic of debate in literature due to potential complications such as blockage, migration, or infection [38, 39]. Naturally, it is challenging to conduct comparative trials or observational studies to evaluate these shunting techniques due to the small sample size, lack of long-term follow-up, and failure to reach statistical significance.

Patient prognosis also is a topic of ongoing debate in the literature. While a review of the literature (Table 1) indicates symptomatic and radiological improvement in all observed patients, full recovery is exceptionally rare. Time to symptom improvement also varies depending on the severity of symptoms and the level and extent of involvement of the spinal cord. A few patients also experienced recurrence, although the cause remains to be investigated. Repeated surgical manipulation seems to provide relief [13]. Patients often require physical rehabilitation to improve day-to-day function and quality of life.

## Conclusions

Delayed spinal arachnoiditis associated with syringomyelia is a rare complication of aneurysmal SAH. Risk factors include female gender, aneurysm in the posterior circulation, and advanced age. Primary treatment includes laminectomy accompanied by adhesion microlysis and shunt placement. Rehabilitation may be necessary in most patients, as full symptomatic recovery may not be achieved.

## Timeline

**Fig. 5:**
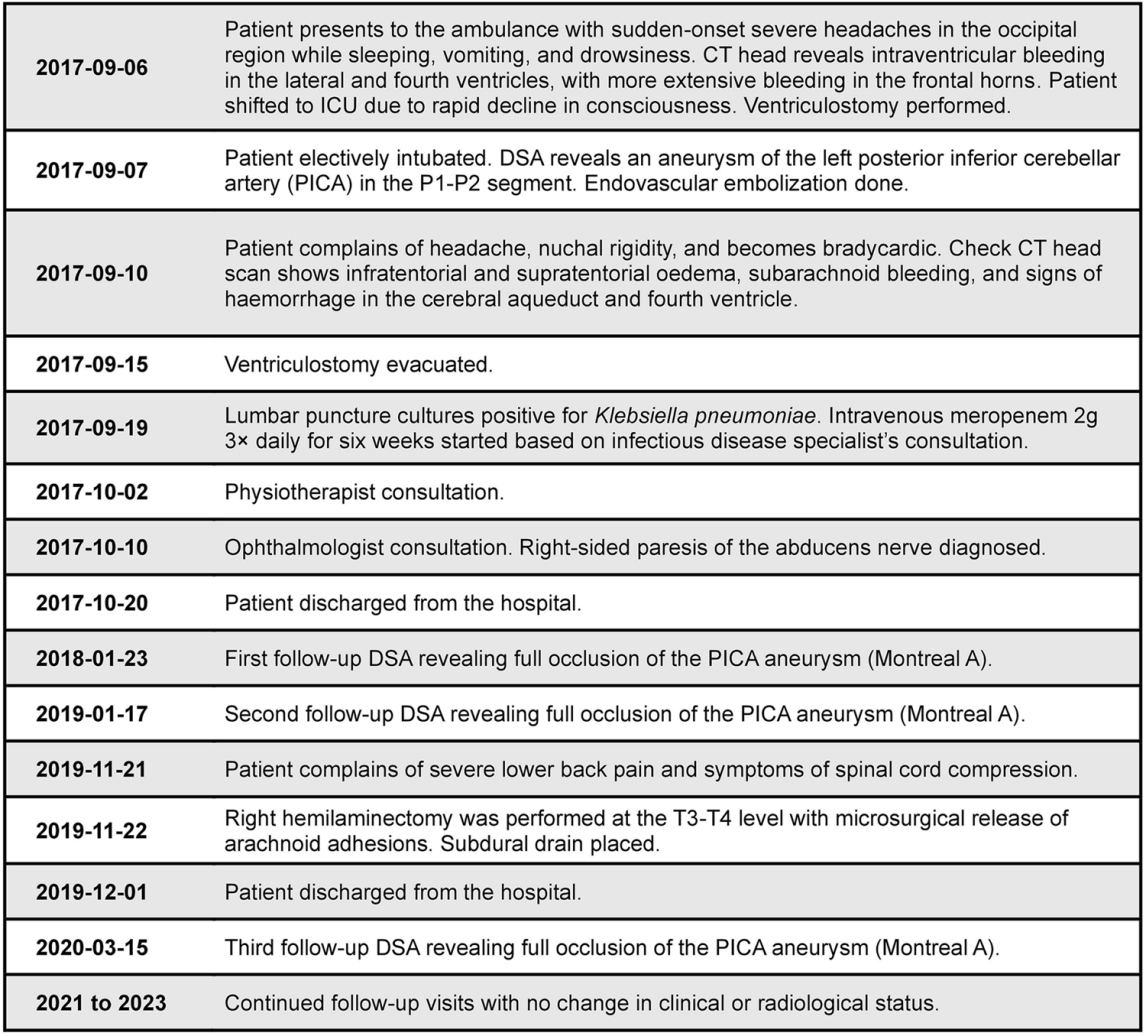
Timeline of key events for the present case report.

A timeline of the key events has been summarized in Fig. 5.

## Data Availability

The data that support the findings of this study are available from the corresponding author upon reasonable request.
